# First Case Report of Acquired Copper Deficiency Following Revisional Single Anastomosis Duodeno-Ileal Bypass with Sleeve Gastrectomy (SADI-S) Leading to Severe Pancytopenia with Refractory Anemia

**DOI:** 10.1007/s11695-020-04916-3

**Published:** 2020-08-28

**Authors:** Alyaa Abusabeib, Walid El Ansari, Wahiba Elhag

**Affiliations:** 1 Department of Bariatric Surgery/Bariatric Medicine, Hamad General Hospital, 3050 Doha, Qatar; 2 Department of Surgery, Hamad General Hospital, 3050 Doha, Qatar; 3 College of Medicine, Qatar University, Doha, Qatar; 4 Schools of Health and Education, University of Skovde, Skövde, Sweden

## Background

Copper, a largely available trace element in the human body, is a cofactor in many enzymatic reactions that are vital for the functioning of the hematologic, vascular, skeletal, antioxidant, and neurologic systems [1, 2]. It is absorbed mainly in the stomach and proximal duodenum [3]. Copper deficiency is extremely unusual in healthy individuals [4].

Bariatric surgical procedures cause anatomical changes of the gastrointestinal tract that could lead to hypocupremia and/or predispose patients to a range of nutritional deficiencies that can lead to anemia, osteoporosis, and protein malnutrition [5]. Thus, without appropriate supplementation of a range of micro- and macronutrients post-bariatric procedures, patients might develop such deficiencies.

Single anastomosis duodeno-ileal bypass with sleeve gastrectomy (SADI-S) is a relatively recent bariatric surgical procedure in which sleeve gastrectomy is followed by end-to-side duodeno-ileal diversion [6]. The elimination of one anastomosis results in decreased surgery time and possibly less surgery-related complications [7]. Whilst SADI-S has significant weight loss and positive metabolic outcomes, malabsorptive effects might occur, e.g., albumin, zinc, folate, vitamins A, D, and E, zinc, and copper [6, 7]. The diagnosis of hypocupremia could be challenging due to its rarity and its similar clinical presentation as vitamin B_12_ deficiency [8].

Published reports of the malnutrition outcomes after SADI-S are extremely rare [9]. An exception is a study of 97 SADI-S patients, where 12% developed copper deficiency at 1 year and 11% at 3 years post-op [10]. We report a case of severe copper deficiency after revisional SADI-S leading to severe pancytopenia with cellular atypia. To the best of our knowledge, this could be the first case report of severe copper deficiency leading to profound hematological abnormalities post-SADI-S.

## Case Report

**Fig. 1:**
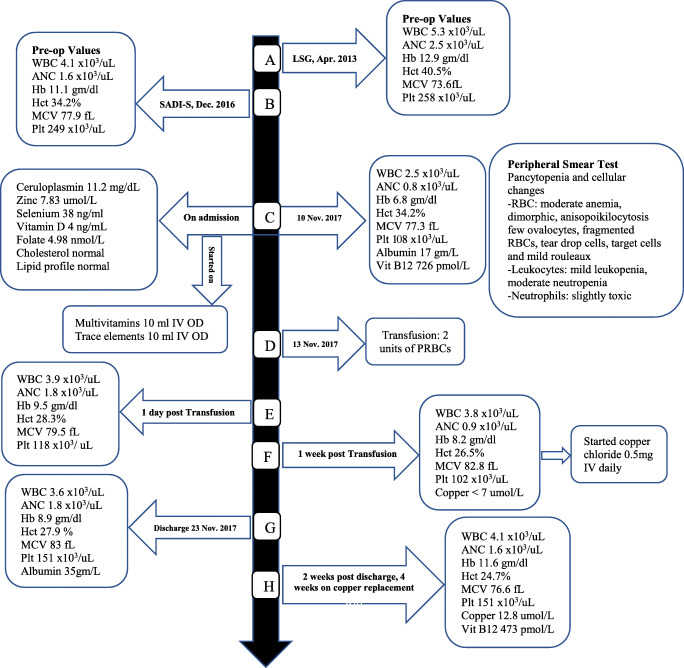
Timeline and sequence of events over 7 years. *LSG* Laparascopic sleeve gastrectomy, *SADI-S* single anastomosis duodeno-ileal bypass with sleeve gastrectomy, *WBC* white cell count (4 × 10^3^/uL–10 × 10^3^/uL), *Hct* hematocrit (36–46%), *MCV* Mean corpuscular volume (83–101 fL), *Hb* hemoglobin (12–15 g/dl), *ANC* absolute neutrophil count (2 × 10^3^/uL–7 × 10^3^/uL), *Plt* platelet (150 × 10^3^/uL–400 × 10^3^/uL), albumin (35–50 g/L), vitamin B_12_ (133–675 pmol/L), ceruloplasmin (20–60 mg/dL), zinc (10.1–16.8 umol/L), selenium (70–150 ng/ml), vitamin D (35-80 ng/mL), folate 4.98 nmol/L (10.4–42.4 nmol/L), *PRBCs* packed red blood cells. *OD* once daily

Figure 1 depicts the sequence of events over 7 years.

A 37-year-old Qatari female presented to our outpatient bariatric clinic (Hamad General Hospital, largest tertiary care institution in Qatar) with a 4-month complaint of generalized fatigability and progressive bilateral lower limbs swelling with occasional numbness of the distal aspects of the lower limbs but no weakness. Her complaints had worsened over time, and were affecting her quality of life and ability to work. She also had frequent episodes of palpitations, exertional shortness of breath, but no chest pain. There was no nausea or vomiting, but she indicated that there was an increase in her bowel motions which had become more greasy. She denied bloody stools abdominal pain, change in appetite, fever, joint pain, or skin changes. She also complained of occasional headaches but no visual changes. Past medical history was remarkable for T2DM controlled with oral hypoglycemic agents (sitagliptin 50 mg and metformin 1000) and basal insulin, hypertension controlled by antihypertensives (valsartan 160 mg and hydrochlorothiazide 12.5 mg), bronchial asthma with recurrent exacerbations, primary infertility, and severe obesity.

The patient had undertaken laparoscopic sleeve gastrectomy (LSG) on April 2013 (Fig. 1A) as her weight then was 162 kg (BMI 57 kg/m^2^). After the LSG, her minimum post-operative weight was 90 kg, the T2DM and hypertension resolved within the first year post-operatively and she was off medications for both conditions. Moreover, her asthma exacerbations became minimal. Weight regain started on the second year post-LSG until she reached 118 kg (BMI 42 kg/m^2^). Hence, 3 years after her initial LSG, in December 2016, she underwent revisional laparoscopic SADI-S. Eleven months after the SADI-S (November 2017), she presented to our bariatric surgery clinic with the abovementioned complaints, claimed to be adherent to the multivitamin with minerals daily tablets and iron supplements, and denied smoking or alcohol consumption.

Upon examination, she appeared vitally stable, weighed 93.6 kg (BMI 35.6 kg/m^2^), with normal cardiovascular, chest, and abdominal examinations, and no neurological deficits. She appeared pale and had significant bilateral lower limb edema (Grade 3), and was admitted for inpatient assessment and further management.

Laboratory results upon admission showed microcytic hypochromic anemia, leucopenia, thrombocytopenia, hypoalbuminemia, and high level of vitamin B_12_ (Fig. 1C). She was started on IV multivitamins and trace elements, thiamine IV, and high-protein diet, and eventually required total parental nutrition until laboratory results became available. Peripheral blood smear confirmed pancytopenia with cellular atypia (Fig. 1C). Further investigations revealed low ceruloplasmin, and severe deficiencies of copper, zinc, selenium, vitamin D, and folate but normal cholesterol and lipid profile (Fig. 1C). Hemoglobin electrophoresis showed normal hemoglobin pattern.

For the severe microcytic hypochromic anemia, she received a transfusion of two units of packed red blood cells (Fig. 1D) that resulted in a transient rise in hematocrit count and hemoglobin level (Fig. 1E), followed by a gradual drop of both levels over the following days (Fig. 1F). The pancytopenia progressively improved following the initiation of an additional 0.5 mg of copper chloride IV daily dose after diagnosis of copper deficiency.

Screening for celiac disease was negative and esophagogastroduodenoscopy was normal. She was hospitalized for 2 weeks. At discharge, her blood picture was improving (Fig. 1G). She was prescribed multivitamin and minerals daily tablet, copper gluconate 2 mg daily, selenium 200 mcg bid, vitamin D, calcium and iron supplements, cyanocobalamin 1 mg daily, pancreatic enzymes for suspected pancreatic insufficiency, and high-protein supplement. At follow-up, 2 weeks after discharge, her laboratory results showed a normal level of serum copper, resolution of the leukopenia, neutropenia, and thrombocytopenia, and improvement of microcytic hypochromic anemia (Fig. 1H). She reported significant improvements in her symptoms and better quality of life.

## Discussion

We report a case report of severe copper deficiency leading to hematological abnormalities post-SADI-S. The mechanism/s by which copper deficiency causes anemia are multifactorial. Copper and iron interact through ceruloplasmin, a copper-dependent oxidase that helps to transport iron into the plasma along with transferrin [11]. In addition, hephaestin, a trans-membrane copper containing ferroxidase, helps to export iron from enterocytes into the blood circulation, and as copper is essential to mobilize iron from the liver to the bone marrow where it is consumed, thus copper significantly affects erythropoiesis [11]. These mechanisms and interactions explain how the laboratory findings of copper deficiency and myelodysplastic syndromes mimic each other [11, 12]. Our patient had anemia and leucopenia (mainly neutropenia), in agreement with myelodysplastic syndrome [11, 12]. In addition, she had moderate thrombocytopenia which is less frequently reported in copper deficiency [4].

The patient underwent LSG as a primary weight loss surgery followed by SADI-S as revisional surgery. Weight loss surgery that is characterized by combined restrictive and malabsorptive effects alters copper absorption in the stomach, duodenum, and ilium [13]. In addition, other factors e.g. pre-operative deficiencies, post-surgery food intolerance, changes in taste and eating patterns, and non-adherence to dietary and supplement recommendations could collectively lead to copper deficiency. The literature completely lacks case reports outlining the types and levels of the range of micro- and macronutrient deficiencies after SADI-S. However, copper deficiency has been reported following mainly malabsorptive procedures, e.g., gastric bypass surgery [14, 15], and less frequently after sleeve gastrectomy and duodenal switch [16, 17].

While the neurological examination of our patient was normal, she mentioned a history of occasional episodes of lower limb numbness that might be attributed to the copper deficiency, given that her laboratory results showed normal thiamine and vitamin B_12_ blood levels. Various myelopathies and peripheral neuropathies have been reported with copper deficiency, some of which were severe and irreversible [3, 4, 18, 19]. In addition, although copper deficiency has been observed as a possible cause of ischemic heart disease by significantly promoting major risk factors such as hypercholesterolemia, chronic inflammation, oxidative stress, and glucose intolerance [20], our patient had normal cholesterol and lipid profiles.

Refractory iron deficiency anemia with copper deficiency has been described post-duodenal switch surgery [21]. In our case, the two units of packed red blood cells transfusion resulted in a transient rise in hematocrit count and hemoglobin level (Fig. 1E), followed by a gradual dropping of both, highlighting that among anemic patients, copper deficiency could cause a refractory response to blood transfusion and iron replacement. Hence, prompt diagnosis and treatment of copper deficiency could help to prevent serious complications such as severe pancytopenia with its consequences and neuropathy. Finally, although the patient also had concomitant folate deficiency, however, her peripheral smear showed hypochromic microcytic anemia (as opposed to the macrocytic anemia observed with folate deficiency), suggesting that among post-bariatric surgery patients with concomitant hypocuperimia, that the effects of the copper deficiency on erythropoiesis could be more pronounced than that of the folate.

## Conclusion

Although rare, the current case emphasizes that the bariatric team should be aware that after bariatric procedures such as SADI-S, micronutrient deficiencies could be encountered. In order to avoid complications, early diagnosis, and prompt treatment of copper and other micronutrient deficiencies are critical. Mild neurological symptoms among post-bariatric surgery patients should alert the bariatric team to possible micronutrient/copper deficiency. Findings of anemia, pancytopenia, and cellular atypia warrant extensive assessment and replacement of micronutrients.
